# Association between triglyceride glucose-waist-adjusted waist index and incident stroke in Chinese adults: a prospective cohort study

**DOI:** 10.3389/fnut.2025.1612864

**Published:** 2025-07-01

**Authors:** Yaqin Ye, Zhenyi He, Jingguo Wu, Junlin Wu, Yanbing Liang

**Affiliations:** Department of General Practice Medicine, The First Affiliated Hospital, Sun Yat-sen University, Guangzhou, China

**Keywords:** stroke, TYG-WWI, nonlinear association, insulin resistance, smooth curve fitting

## Abstract

**Objective:**

Current literature lacks evidence on the association between the triglyceride glucose-weight-adjusted waist index (TyG-WWI) and incident stroke. Therefore, this study aims to elucidate the relationship between TyG-WWI and stroke risk in a nationally representative cohort of middle-aged and elderly Chinese adults by: (1) Quantitatively assessing its prospective association with incident stroke, and (2) Investigating potential nonlinear relationships and inflection points.

**Methods:**

This national longitudinal study examined data from five waves of the CHARLS (2011–2020). TyG-WWI served as the key exposure variable, while stroke incidence, as determined by physician diagnosis, was the principal outcome of interest. Associations were evaluated using Cox proportional hazards models alongside nonlinear analyses, with careful adjustment for a range of covariates. To further ensure the reliability of our findings, we performed a series of sensitivity and subgroup analyses.

**Results:**

In this study, we investigated the association between TyG-WWI and stroke risk. The results demonstrated a significant positive correlation between TyG-WWI quartiles and stroke risk, with HRs of 1.35 (95% CI 1.08–1.69, *p* = 0.0094), 1.38 (95% CI 1.09–1.73, *p* = 0.0069), and 1.42 (95% CI 1.09–1.85, *p* = 0.0086) for Q2, Q3, and Q4, respectively, compared to Q1 in Model III (P for trend = 0.0158). A two-piecewise linear regression model revealed an inflection point of TyG-WWI at 43.32, with a significant association between TyG-WWI and stroke risk only above this point (HR = 1.03, 95% CI 1.01–1.05, *p* = 0.0008). Stratified analysis indicated a more robust association in non-hypertensive individuals (HR = 1.94, 95% CI 1.29–2.91, *p* = 0.0014 in Q4) and a significant interaction for hypertension status (P for interaction = 0.0493).

**Conclusion:**

In conclusion, our study reveals a significant association between TyG-WWI and stroke risk in the middle-aged and elderly population in China, with stroke risk increasing progressively across higher TyG-WWI quartiles. Notably, this association was significantly more pronounced in non-hypertensive individuals. Furthermore, an inflection point indicates a stronger association beyond this threshold.

## 1 Introduction

Stroke continues to be a leading cause of mortality and long-term disability globally (1, 2), with a particularly escalating burden observed in aging populations (3, 4). Recent analyses indicate a rising incidence of stroke-related morbidity and mortality, driven in part by the demographic trend of an aging population (5, 6). This demographic shift underscores the importance of addressing traditional risk factors, including obesity (7), insulin resistance (8, 9), and central adiposity (10, 11), which are critical contributors to stroke pathogenesis.

Commonly, the weight-adjusted waist index (WWI) is utilized as a standard measure for evaluating obesity-related risk (12). The WWI is posited to offer a refined measure of visceral fat accumulation, operating independently of total adiposity (13). Visceral fat is particularly associated with metabolic disturbances that heighten the risk of cerebrovascular accidents (14, 15). However, these metrics have evident limitations in effectively distinguishing between fat distribution and nuances of metabolic health (16). Thus, reliance on the WWI alone may not sufficiently capture the intricacies surrounding the impact of obesity on stroke risk. Recent discussions emphasize the need for more integrative biomarkers that account for the metabolic distress that contributes to cardiovascular conditions, highlighting dyslipidemia, impaired glucose metabolism, and visceral adiposity as central players in atherogenesis and cerebrovascular events (16, 17). Insulin resistance is an independent risk factor for ischemic stroke (15, 18). The TyG index has been identified as a reliable alternative biomarker of insulin resistance (19). Studies have shown that patients with insulin resistance, as well as those exhibiting high levels of triglycerides, are at a notably greater risk of experiencing acute ischemic strokes, with some research indicating that this risk can be more than double in individuals with high insulin concentrations within nondiabetic populations (20, 21). Nevertheless, there are certain limitations to the use of the TyG index as a marker for cardiovascular disease (22). The correlation of insulin resistance with elevated triglyceride levels highlights the urgency for comprehensive assessments that integrate metrics such as the TyG-WWI (23, 24). A thorough appraisal of metabolic factors, including the role of insulin resistance and the implications of central obesity for cardiovascular health, is therefore crucial for developing effective preventive strategies against stroke (14, 15, 25).

The TyG-WWI, which combines triglyceride levels and the waist-to-height ratio, is a novel measure that shows promise in capturing metabolic derangements affecting stroke outcomes and could strengthen the identification of high-risk populations and improve the implementation of targeted prevention strategies; however, it remains underutilized in stroke research and should be integrated into stroke epidemiology (17, 26).

Leveraging data from the China Health and Retirement Longitudinal Study (CHARLS), a nationally representative cohort, this study addressed a critical gap in evidence. While insulin resistance (reflected by the TyG index) and central adiposity (reflected by the WWI) are established risk factors for stroke, robust evidence on their combined metric, the TyG-WWI, remains scarce. Therefore, this longitudinal study aimed to:(1) Quantitatively assess the prospective association between baseline TyG-WWI and incident stroke risk. (2) Specifically investigate potential nonlinear relationships in this association and identify any inflection points.

## 2 Methods

### 2.1 Study design

This nationwide prospective cohort study leverages longitudinal data from five waves of the China Health and Retirement Longitudinal Study (CHARLS) (27) spanning from 2011 to 2020. The primary exposure variable was the triglyceride–glucose waist-type-like index (TyG-WWI), whereas the incidence of stroke, recorded as a binary outcome (stroke = 1, no stroke = 0), was the main outcome of interest. Participants with preexisting stroke conditions at baseline, those with incomplete data on key variables, including TyG-WWI components, and individuals lost to follow-up were excluded from the analysis to ensure data integrity and reliability.

### 2.2 Data sources and study population

Data for this research were obtained from the China Health and Retirement Longitudinal Study (CHARLS) (27), a nationally representative cohort study focused on the health, economic, and social conditions of China’s middle-aged and elderly population. The CHARLS cohort was formed through a multistage stratified sampling method, recruiting participants from 450 communities across 150 counties in 28 provinces, with 10,257 households included in the initial survey. The baseline survey took place from June 2011 to March 2012 and covered individuals aged 45 years and older. Follow-up surveys were conducted every 2 years. The study was approved by the Biomedical Ethics Review Board of Peking University (IRB00001052-11015), and all participants provided written informed consent. The datasets used and analyzed in this study are available on the CHARLS project website.^1^

**Figure 1:**
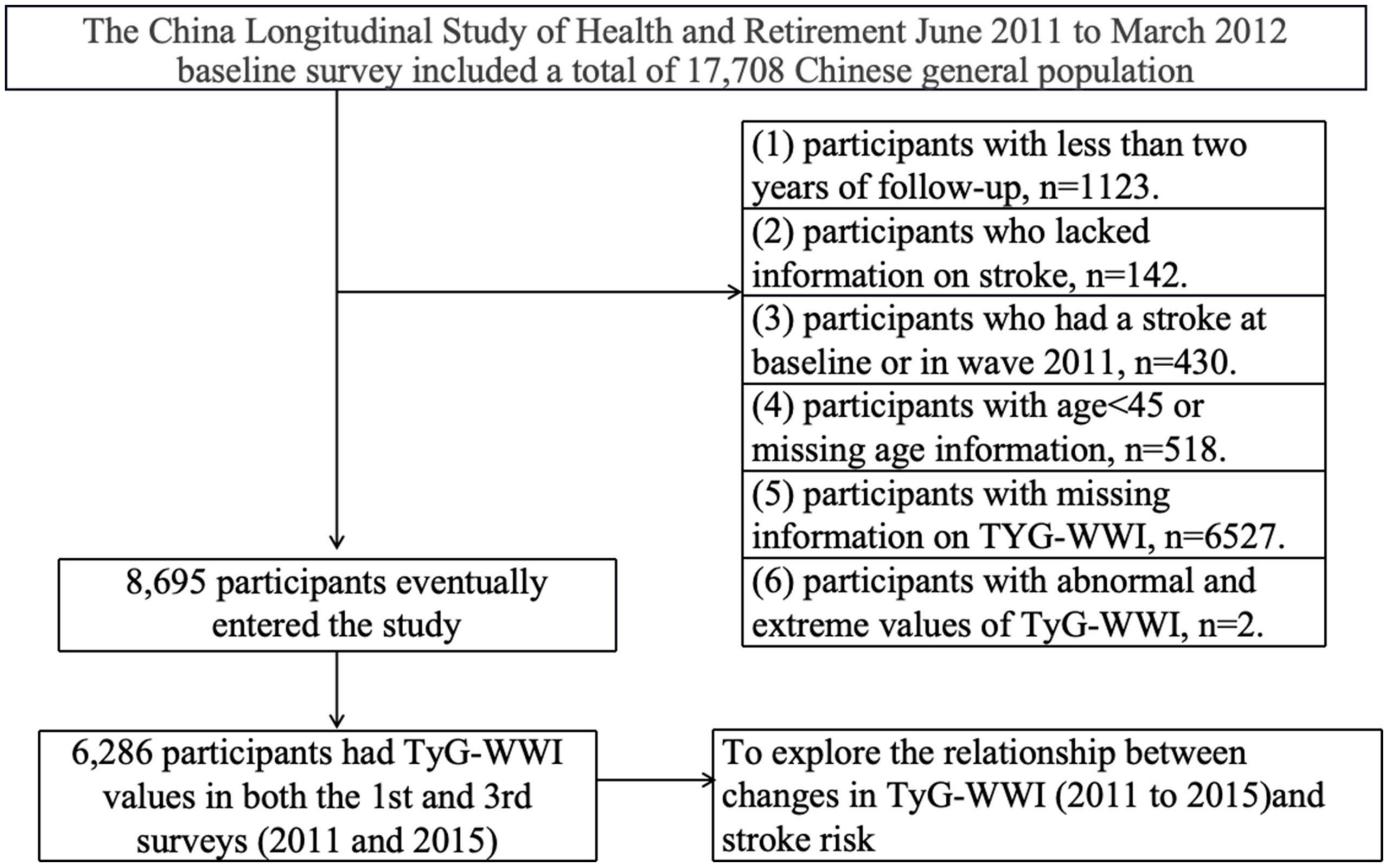
Flowchart illustrating the selection process of the study participants.

This investigation incorporated only participants with complete datasets, excluding those with follow-up periods shorter than 2 years (*n* = 1,123), missing stroke information (*n* = 142), a history of stroke at baseline or in wave 2011 (*n* = 430), age below 45 years or missing age data (*n* = 518), incomplete TyG-WWI information (*n* = 6,527), and abnormal or extreme TyG-WWI values (*n* = 2), resulting in a final study population of 8,695 participants (Figure 1).

## 3 Variables

### 3.1 TYG-WWI

Participants’ fasting blood glucose, triglyceride, weight, and waist circumference were measured (28). The TyG-WWI was calculated via the following steps: TyG calculation: TyG was determined via the following formula: TyG = TyG = ln [triglycerides (mg/dl) × fasting blood glucose (mg/dl)]/2 (29). This formula integrates two critical metabolic parameters, providing a composite measure of insulin resistance. WWI calculation: WWI was computed as waist circumference (cm) divided by the square root of weight (kg), i.e., WWI = waist circumference (cm)/√weight (kg). This index reflects abdominal obesity relative to overall body weight. TyG-WWI Calculation: The TyG-WWI was obtained by multiplying TyG and WWI, i.e., TyG-WWI = TyG × WWI (30). This combined metric offers a more comprehensive assessment of metabolic and adiposity factors (31).

### 3.2 Stroke

The primary endpoint of this study was stroke, which was determined mainly through physician diagnosis. In this study, when a participant reported a stroke during the follow-up, the follow-up time was recorded as the time at which the event occurred. For participants who did not report a stroke during the follow-up period, the follow-up duration was determined on the basis of the interval between the baseline assessment and the final survey date (32, 33).

### 3.3 Covariates

Covariates were selected based on prior research and clinical relevance to stroke risk and insulin resistance. Age, sex, smoking status, drinking status, hypertension, and diabetes mellitus are widely acknowledged as factors that may exert a notable influence on health and disease (32, 34, 35). Chronic comorbidities, including cancer (36), chronic kidney disease (CKD) (37), and chronic lung disease (CLD) (38), were included as covariates due to their potential influence on metabolic status, systemic inflammation, and vascular health. Lipid profiles, including high-density lipoprotein cholesterol (HDL-c), low-density lipoprotein cholesterol (LDL-c), and total cholesterol (TC), were included due to their strong associations with metabolic dysfunction and cardiovascular risk (32, 35). As commonly used biomarkers of systemic inflammation, C-reactive protein (CRP) and white blood cell count (WBC) have been closely linked to insulin resistance, visceral adiposity, and the development of cardiovascular and cerebrovascular diseases (39, 40). Renal function–related measures, including estimated glomerular filtration rate (eGFR) (41), cystatin C (CYSC), and uric acid (UA), were included as covariates because impaired kidney function has been associated with insulin resistance and increased risk of cerebrovascular events. EGFR and elevated cystatin C levels are linked to higher stroke risk, while elevated UA levels are associated with both insulin resistance and poor stroke outcomes (42, 43). Hemoglobin A1c (HBA1c) is an observational indicator of long-term glycemic control, which is related to metabolic health and vascular complications (32, 44). Hemoglobin (HGB) levels were considered to reflect oxygen-carrying capacity and anemia status, both of which may influence stroke risk (45, 46). Platelet count (PLT) was included in the analysis because platelets play a critical role in thrombosis and inflammatory responses, both of which are central components of stroke pathogenesis (47).

### 3.4 Statistical analyses

All the statistical analyses were performed via R language (version 4.4.0) and Empower (R) software (version 6.0). A two-sided *p* value <0.05 was considered statistically significant. Initially, missing values of other variables were handled through multiple imputation by chained equations (MICE) using the R package mice (48). Then, participants were stratified by TyG-WWI quartiles. Data were characterized as means/SDs or medians/IQRs for continuous variables and frequencies/percentages for categorical variables. Group comparisons used one-way ANOVA, the Kruskal-Wallis test, and the chi-square test. Next, the overall stroke incidence rate and stroke incidence rates across TyG-WWI quartiles (Q1–Q4) were calculated. To assess the association between individual factors and stroke risk, univariate Cox proportional hazards regression analysis was employed, estimating the hazard ratio (HR) and 95% confidence interval (CI) for each factor. Variance Inflation Factors (VIF) for covariates in multivariable models were calculated (Supplementary Table S1). The association of TyG-WWI with stroke risk was assessed using Cox proportional hazard regression models, which estimated adjusted HRs with 95% CIs. Three models were used: model I (unadjusted), model II (adjusted for age and sex), and model III (adjusted for extensive variables like age, sex, drinking/smoking status, cancer, CLD, CKD, hypertension, diabetes, and multiple blood—related parameters). TyG-WWI was analyzed both as a continuous variable (per 1-unit increase) and categorically (quartiles Q1–Q4, with Q1 as reference). A trend test assessed the linear trend across TyG-WWI quartiles by including the quartile rank as a continuous variable. Subsequently, the independent impact of TyG, FPG, and WWI on stroke risk was re-evaluated. Given that TyG-WWI was a continuous variable, generalized additive models (GAM) were used to check for non-linear relationships. If non-linear correlations were found, a two—piecewise linear regression model was applied to calculate the threshold effect of TyG-WWI on stroke incidence based on smoothing plots, with the recursive method automatically determining the inflection point where model likelihood was maximized. We used stratified Cox proportional hazards regression models to evaluate the relationship between TyG-WWI quartiles and stroke risk across different subgroups (age, sex, hypertension status, diabetes status, CKD, drinking status, and smoking status) and conducted interaction tests for each subgroup. Each model was adjusted for confounders but not for the stratification variables. Finally, participants were grouped by TyG-WWI changes from 2011 to 2015 via K-means cluster analysis into Class 1 (low TyG-WWI) and Class 2 (high TyG-WWI). Multivariate logistic regression assessed the link between these groups and stroke risk (32, 49).

## 4 Results

### 4.1 Participant characteristics

The study included 8,965 participants aged ≥45 years from the CHARLS cohort. Baseline characteristics were compared across TyG-WWI quartiles (Q1–Q4), and the results are presented in Table 1. The participants in the higher TyG-WWI quartiles were older, with Q4 having the highest mean age (61.80 ± 9.50 years). There were significant differences in lipid profiles across quartiles, with HDL-c, LDL-c, and TC showing graded reductions from Q1 to Q4. For example, HDL-c decreased from 58.15 ± 15.29 mg/dL in Q1 to 43.75 ± 13.63 mg/dL in Q4. Conversely, blood pressure parameters (SBP/DBP) tended to increase in higher quartiles. The prevalence of hypertension and diabetes mellitus (DM) increased significantly with increasing TyG-WWI quartile, reaching 56.65 and 36.82% in Q4, respectively.

**Table 1:** Baseline characteristics of the participants.

TyG-WWI quartile	Q1 (5.65–48.04)	Q2 (48.04–51.90)	Q3 (51.90–56.11)	Q4 (56.11–80.41)	*P* value
*N*	2,241	2,241	2,241	2,242	
Age (years)	57.22 ± 8.69	58.85 ± 9.16	59.42 ± 8.94	61.80 ± 9.50	<0.001
HDL-c (mg/d)	58.15 ± 15.29	53.40 ± 14.74	49.80 ± 13.90	43.75 ± 13.63	<0.001
LDL-c (mg/d)	109.61 ± 30.69	116.68 ± 31.70	122.41 ± 33.82	117.30 ± 42.23	<0.001
TC (mg/dl)	181.26 ± 34.44	188.93 ± 35.24	197.50 ± 36.74	208.08 ± 42.87	<0.001
HGB (g/d)	14.36 ± 2.21	14.39 ± 2.22	14.37 ± 2.15	14.40 ± 2.22	0.927
CRP (mg/l)	2.37 ± 6.84	2.60 ± 7.99	2.62 ± 7.42	2.91 ± 5.97	0.088
WBC (10^9^/L)	5.99 ± 1.82	6.15 ± 1.90	6.32 ± 1.88	6.51 ± 1.88	<0.001
PLT (10^9^/L)	207.95 ± 72.94	210.45 ± 70.35	212.92 ± 73.45	216.16 ± 74.91	0.001
HbA1 (%)	5.07 ± 0.48	5.14 ± 0.53	5.25 ± 0.66	5.61 ± 1.21	<0.001
CYSC (mg/l)	1.01 ± 0.22	1.01 ± 0.30	1.01 ± 0.27	0.99 ± 0.27	0.013
UA (mg/dl)	4.35 ± 1.15	4.38 ± 1.22	4.48 ± 1.26	4.59 ± 1.33	<0.001
eGFR (mL/min/1.73 m^2^)	100.13 ± 14.24	97.73 ± 14.78	96.26 ± 15.31	93.45 ± 16.85	<0.001
DBP	73.22 ± 12.15	74.23 ± 11.84	76.14 ± 12.10	77.65 ± 12.24	<0.001
SBP	124.33 ± 20.12	126.48 ± 19.78	130.91 ± 21.40	135.98 ± 22.51	<0.001
Sex					<0.001
Female	789 (35.21%)	1,037 (46.27%)	1,329 (59.30%)	1,661 (74.09%)	
Male	1,452 (64.79%)	1,204 (53.73%)	912 (40.70%)	581 (25.91%)	
Drinking status					<0.001
Never	1,149 (51.27%)	1,291 (57.61%)	1,448 (64.61%)	1,596 (71.19%)	
Ever	165 (7.36%)	199 (8.88%)	207 (9.24%)	156 (6.96%)	
Current	927 (41.37%)	751 (33.51%)	586 (26.15%)	490 (21.86%)	
Smoking status					<0.001
Never	1,075 (47.97%)	1,277 (56.98%)	1,471 (65.64%)	1,661 (74.09%)	
Ever	971 (43.33%)	745 (33.24%)	581 (25.93%)	415 (18.51%)	
Current	195 (8.70%)	219 (9.77%)	189 (8.43%)	166 (7.40%)	
Cancer					0.414
No	2,223 (99.20%)	2,226 (99.33%)	2,223 (99.20%)	2,217 (98.88%)	
Yes	18 (0.80%)	15 (0.67%)	18 (0.80%)	25 (1.12%)	
CLD					0.008
No	2057 (91.79%)	1992 (88.89%)	2035 (90.81%)	2017 (89.96%)	
Yes	184 (8.21%)	249 (11.11%)	206 (9.19%)	225 (10.04%)	
CKD					0.500
No	2,100 (93.71%)	2,123 (94.73%)	2,113 (94.29%)	2,118 (94.47%)	
Yes	141 (6.29%)	118 (5.27%)	128 (5.71%)	124 (5.53%)	
Hypertension					<0.001
No	1,646 (73.45%)	1,488 (66.40%)	1,271 (56.72%)	972 (43.35%)	
Yes	595 (26.55%)	753 (33.60%)	970 (43.28%)	1,270 (56.65%)	
Diabetes					<0.001
No	2,108 (94.07%)	2040 (91.03%)	1876 (83.71%)	1,439 (64.18%)	
Yes	133 (5.93%)	201 (8.97%)	365 (16.29%)	803 (35.82%)	
Stroke					<0.001
No	2,110 (94.15%)	2042 (91.12%)	2012 (89.78%)	1967 (87.73%)	
Yes	131 (5.85%)	199 (8.88%)	229 (10.22%)	275 (12.27%)	

The data in the table are presented as the means ± SDs or *N* (%). *p* values were calculated via the Kruskal–Wallis rank sum test for continuous variables and Fisher’s exact probability test for count variables when the theoretical number was less than 10. SD, standard deviation; *N*, number; HDL-c, high-density lipoprotein cholesterol; LDL-c, low-density lipoprotein cholesterol; TC, total cholesterol; HGB, hemoglobin concentration; CRP, C-reactive protein; WBC, white blood cell in thousands; PLT, platelets; HBA1c, hemoglobin A1c; CYSC, cystatin C; UA, uric acid; eGFR, estimated glomerular filtration rate; SBP, systolic blood pressure; DBP, diastolic blood pressure; CLD, chronic lung diseases; CKD, chronic kidney diseases.

### 4.2 Incidence rate of stroke

**Figure 2:**
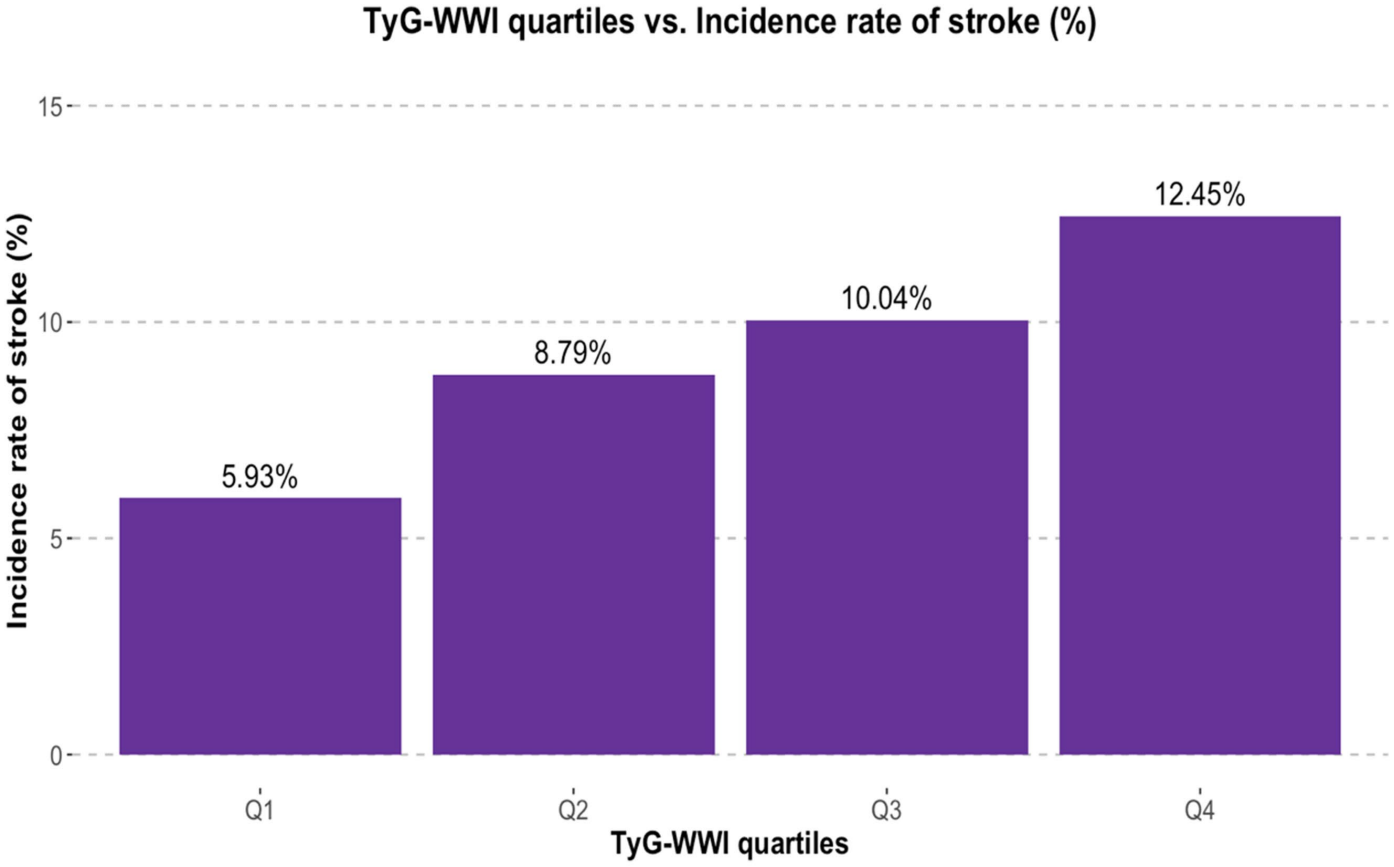
Bar chart representing the incidence of stroke across different quartiles of the TyG-WWI.

Among the 8,965 participants, the overall stroke incidence rate was 9.30% (95% CI 8.70–9.90), or 117.61 per 10,000 person-years, as shown in Table 2. Stratified by TyG-WWI quartiles, the incidence rates were 5.84% (71.9 per 10,000) for Q1, 8.88% (112 per 10,000) for Q2, 10.20% (129 per 10,000) for Q3, and 12.30% (161 per 10,000) for Q4. The *p* value for the trend was 0.083, indicating a nearly significant increase in stroke incidence with increasing TyG-WWI quartiles. The bar chart in Figure 2 visually presents an upward trend in stroke incidence as TyG-WWI quartiles increase. Specifically, the incidence rate rises from 5.93% in Q1 to 12.45% in Q4, indicating a potential positive correlation between TyG-WWI levels and stroke risk.

**Table 2:** Incidence rates of stroke.

TyG-WWI	Participants (*n*)	Stroke events (*n*)	Incidence rate (95% CI) (%)	Per 10,000 person-year
Total	8,965	834	9.30 (8.70–9.90)	117.61
Q1 (5.65–48.04)	2,242	133	5.84 (4.87–6.81)	71.9
Q2 (48.04–51.90)	2,241	199	8.88 (7.70–10.10)	112
Q3 (51.90–56.11)	2,241	229	10.20 (8.96–11.50)	129
Q4 (56.11–80.41)	2,241	275	12.30 (10.9–13.60)	161
P for trend			0.083	

TyG-WWI, triglyceride glucose-weight-adjusted waist index; CI, confidence.

### 4.3 Stroke risk factors identified through univariate cox proportional hazards regression

Supplementary Table S2 presents the results of the univariate Cox proportional hazards regression analysis, which identified several factors significantly associated with stroke risk. Age was significantly associated with a HR of 1.03 (95% CI 1.03, 1.04; *p* < 0.0001) per year increase. Male sex was associated with a greater risk, although the difference was not statistically significant (HR = 1.11, 95% CI 0.97, 1.27; *p* = 0.1249). Ever drinkers presented a significantly greater risk than never drinkers did (HR = 1.49, 95% CI 1.19, 1.86; *p* = 0.0006), whereas current drinkers did not present a significant difference. Current smokers had a significantly greater risk than never smokers did (HR = 1.63, 95% CI 1.31, 2.02; *p* < 0.0001), whereas the risk did not significantly differ across smokers. A history of cancer, CLD, or CKD showed nonsignificant or weak associations with stroke risk. Hypertension and diabetes were strongly associated with increased stroke risk (HR = 2.48, 95% CI 2.16, 2.84, *p* < 0.0001 and HR = 1.41, 95% CI 1.20, 1.67, *p* < 0.0001, respectively). HDL-c was inversely associated with stroke risk (HR = 0.99, 95% CI 0.98, 0.99; *p* < 0.0001), whereas LDL-c and TC showed weak but significant positive associations. Other significant factors included HGB, CRP, WBC, HBA1c, CYSC, UA, and eGFR. Additionally, TyG-WWI showed a significant positive association with stroke risk, with a HR of 1.04 (95% CI1.03, 1.05; *p* < 0.0001) per unit increase.

### 4.4 Association of TyG-WWI with stroke risk

The relationship between the TyG-WWI and the risk of stroke was assessed via Cox proportional hazards regression models (Table 3). In Model I (unadjusted), each unit increase in the TyG-WWI was associated with a 4% increase in stroke risk (HR = 1.04, 95% CI 1.03–1.05; *p* < 0.0001). After adjusting for age and sex in Model II, the HR remained significant (HR = 1.03, 95% CI 1.02–1.05, *p* < 0.0001). In Model III, which included additional covariates such as drinking status, smoking status, cancer status, CLD status, CKD status, hypertension status, diabetes status, HDL, LDL, TC, HGB, CRP, WBC, PLT, HBA1c, CYSC, UA, and eGFR, the association remained statistically significant, with an HR of 1.01 (95% CI 1.00–1.03, *p* = 0.0433).

**Table 3:** Association of TyG-WWI with stroke risk.

Variable	Statistics	Model I (HR., 95%CI) *P*	Model II (HR., 95%CI) *P*	Model III (HR., 95%CI) *P*
TyG-WWI	51.91 ± 7.72	1.04 (1.03, 1.05) < 0.0001	1.03 (1.02, 1.05) < 0.0001	1.01 (1.00, 1.03) 0.0433
TyG-WWI quartile				
Q1 (5.65–48.04)	2,241 (25.00%)	Ref	Ref	Ref
Q2 (48.04–51.90)	2,241 (25.00%)	1.56 (1.25, 1.94) < 0.0001	1.53 (1.22, 1.91) 0.0002	1.35 (1.08, 1.69) 0.0094
Q3 (51.90–56.11)	2,241 (25.00%)	1.79 (1.44, 2.21) < 0.0001	1.79 (1.44, 2.23) < 0.0001	1.38 (1.09, 1.73) 0.0069
Q4 (56.11–80.41)	2,242 (25.01%)	2.26 (1.83, 2.78) < 0.0001	2.20 (1.77, 2.74) < 0.0001	1.42 (1.09, 1.85) 0.0086
P for trend		<0.0001	<0.0001	0.0158

Model I: We did not adjust for other covariates.

Model II: We adjust for age and sex.

Model III: We adjusted for age, sex, drinking status, smoking status, cancer, CLD, CKD, hypertension, diabetes, HDL, LDL, TC, HGB, CRP, WBC, PLT, HBA1c, CYSC, UA, and eGFR.

HR, hazard ratio; Ref, reference; CI, confidence.

For the quartile analysis, compared with that of Q1 (reference), the risk of stroke increased progressively across higher quartiles. In Model III, Q2 had an HR of 1.35 (95% CI 1.08–1.69, *p* = 0.0094), Q3 had an HR of 1.38 (95% CI 1.09–1.73, *p* = 0.0069), and Q4 had an HR of 1.42 (95% CI 1.09–1.85, *p* = 0.0086). The *p* value for trend was significant in Model III (*p* = 0.0158).

### 4.5 Multivariable cox proportional hazards regression to assess the impact of TyG, FPG, and WWI on stroke risk

This study examined the associations of TG, FPG, TyG, and WWI with stroke risk via three Cox proportional hazards regression models (Supplementary Table S3). In Model a, TG was not significantly associated with stroke risk (HR = 1.00, 95% CI 1.00–1.00; *p* = 0.622). Model b revealed a significant association between TyG score and stroke risk (HR = 2.04, 95% CI 1.37–3.04; *p* = 0.0005). Model c revealed no significant relationship between FBG and stroke risk (HR = 1.00, 95% CI 1.00–1.00; *p* = 0.3340). Model d revealed no significant association between the WWI (kg/m^2^) and stroke risk (HR = 1.03, 95% CI 0.97–1.10; *p* = 0.2723). These results highlight TyG as a significant independent predictor of stroke risk, whereas TG, FBG, and WWI individually do not show significant associations with stroke risk in these models.

### 4.6 Nonlinear associations between risk factors and stroke

**Figure 3:**
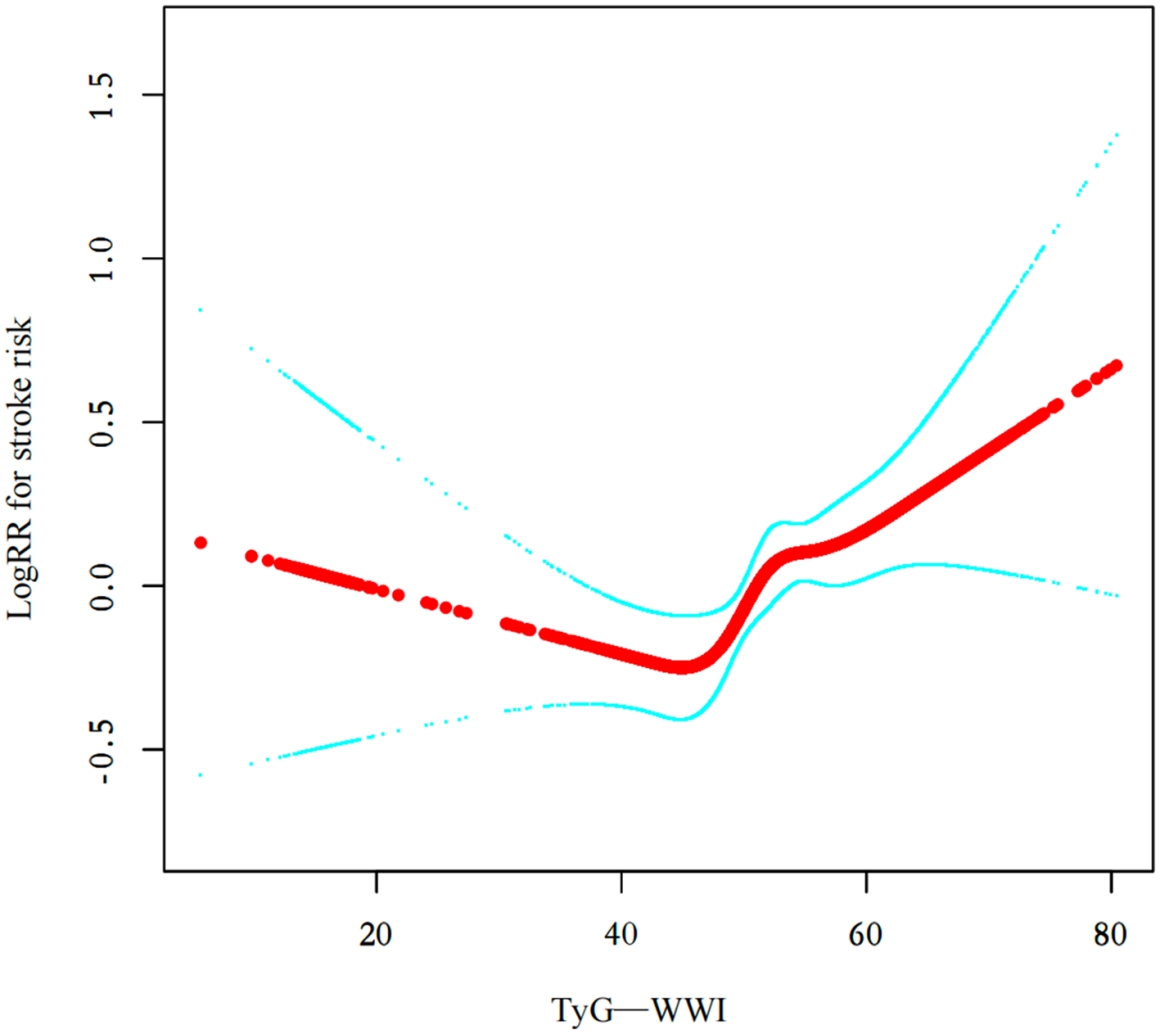
Nonlinear associations between risk factors and stroke. Curve plots demonstrating the nonlinear relationship between the TyG-WWI and stroke risk among all participants adjusted for various factors, including age, sex, drinking status, smoking status, cancer status, CLD, CKD, hypertension, diabetes, HDL, LDL, TC, HGB, CRP, WBC, PLT, HBA1c, CYSC, UA, and eGFR.

In this study, generalized additive models (GAMs) were employed to explore the non-linear association between TyG-WWI and stroke incidence, given that TyG-WWI is a continuous variable (Figure 3). After adjusting for multiple factors, including age, sex, drinking status, smoking status, cancer, CLD, CKD, hypertension, diabetes, HDL, LDL, TC, HGB, CRP, WBC, PLT, HBA1c, CYSC, UA, and eGFR, a non-linear relationship between TyG-WWI and stroke incidence was also observed. By using a two-piecewise linear regression model, we calculated that the inflection point of TyG-WWI was 43.32 (Log- likelihood ratio test *p* = 0.009). Left of the inflection point, TyG-WWI showed a non-significant associated with stroke risk (HR = 0.99, 95% CI 0.97–1.01, *p* = 0.2153). Right of the inflection point, TyG-WWI was significantly associated with increased stroke risk (HR = 1.03, 95% CI 1.01–1.05, *p* = 0.0008) (Table 4).

**Table 4:** Results of the two-piecewise linear regression model.

Outcome: incident stroke	HR (95%CI)	*P* value
Fitting model by standard linear regression	1.01 (1.00, 1.03)	0.0433
Inflection points of TyG-WWI	43.32	
Effect 1 below K	0.99 (0.97, 1.01)	0.2153
Effect 2 above K	1.03 (1.01, 1.05)	0.0008
P for log-likelihood ratio test		0.009

Adjusted for age, sex, drinking status, smoking status, cancer, CLD, CKD, hypertension, diabetes, HDL, LDL, TC, HGB, CRP, WBC, PLT, HBA1c, CYSC, UA, and eGFR.

### 4.7 Subgroup analysis

The Table 5 presents a stratified analysis of the associations between TyG-WWI and the risk of stroke across different characteristics and tests for interaction. Overall, the risk of stroke generally increases with higher TyG-WWI quartiles in most subgroups, though the strength of the associations varies. Notably, a significant interaction is observed for hypertension status (P for interaction = 0.0493), with the significant positive correlation seen in non-hypertensive individuals (HR = 1.94, 95% CI: 1.29–2.91, *p* = 0.0014 in Q4). Other stratification variables, including sex, diabetes, CKD, drinking status, and smoking status, do not show significant interactions (P for interaction values all >0.05).

**Table 5:** Stratified associations between TyG-WWI and stroke by age, sex, hypertension status, diabetes status, CKD, smoking status, and drinking status.

Characteristics	*N*	TyG-WWI				*P* for interaction
		Q1 (5.65–48.04) (HR., 95%CI) *P*	Q2 (48.04–51.90) (HR., 95%CI) *P*	Q3 (51.90–56.11) (HR., 95%CI) *P*	Q4 (56.11–80.41) (HR., 95%CI) *P*	
Age (years)						0.6989
<60	4,911	Ref	1.31 (0.95, 1.80) 0.1023	1.34 (0.96, 1.88) 0.0819	1.53 (1.03, 2.27) 0.0331	
60–69	2,681	Ref	1.71 (1.15, 2.55) 0.0080	1.64 (1.09, 2.45) 0.0168	1.58 (1.00, 2.49) 0.0480	
≥70	1,373	Ref	0.95 (0.54, 1.65) 0.8495	1.01 (0.58, 1.78) 0.9599	1.16 (0.64, 2.10) 0.6272	
Sex						0.7968
Female	4,816	Ref	1.36 (0.94, 1.98) 0.1044	1.25 (0.87, 1.80) 0.2346	1.39 (0.95, 2.04) 0.0895	
Male	4,149	Ref	1.30 (0.98, 1.74) 0.0684	1.45 (1.07, 1.97) 0.0167	1.45 (0.98, 2.16) 0.0634	
Hypertension						0.0493
No	5,377	Ref	1.47 (1.07, 2.02) 0.0177	1.57 (1.11, 2.21) 0.0103	1.94 (1.29, 2.91) 0.0014	
Yes	3,588	Ref	1.19 (0.87, 1.64) 0.2764	1.17 (0.85, 1.60) 0.3398	1.12 (0.79, 1.59) 0.5083	
Diabetes						0.7870
No	7,463	Ref	1.40 (1.10, 1.77) 0.0057	1.42 (1.10, 1.83) 0.0066	1.54 (1.14, 2.09) 0.0049	
Yes	1,502	Ref	1.09 (0.51, 2.35) 0.8174	1.18 (0.60, 2.33) 0.6383	1.08 (0.55, 2.13) 0.8173	
CKD						0.4286
No	8,454	Ref	1.34 (1.06, 1.69) 0.0159	1.41 (1.11, 1.79) 0.0054	1.44 (1.09, 1.89) 0.0100	
Yes	511	Ref	1.69 (0.75, 3.83) 0.2064	0.98 (0.40, 2.41) 0.9610	1.63 (0.63, 4.21) 0.3126	
Drinking status						0.2661
Never drinker	5,484	Ref	1.40 (1.01, 1.94) 0.0406	1.31 (0.94, 1.81) 0.1063	1.47 (1.04, 2.09) 0.0295	
Ever drinker	727	Ref	0.62 (0.31, 1.21) 0.1591	0.72 (0.36, 1.42) 0.3402	0.70 (0.29, 1.66) 0.4125	
Current drinker	2,754	Ref	1.56 (1.08, 2.24) 0.0166	1.82 (1.24, 2.69) 0.0024	1.75 (1.07, 2.87) 0.0255	
Smoking status						0.4039
Never smoker	5,484	Ref	1.32 (0.95, 1.84) 0.0960	1.19 (0.85, 1.65) 0.3147	1.34 (0.94, 1.92) 0.1046	
Ever smoker	2,712	Ref	1.62 (1.13, 2.32) 0.0090	1.82 (1.23, 2.69) 0.0026	1.83 (1.13, 2.99) 0.0150	
Current smoker	769	Ref	0.82 (0.43, 1.55) 0.5356	1.14 (0.61, 2.12) 0.6885	0.95 (0.44, 2.08) 0.9059	

The above model was adjusted for age, sex, drinking status, smoking status, cancer, CLD, CKD, hypertension, diabetes, HDL, LDL, TC, HGB, CRP, WBC, PLT, HBA1c, CYSC, UA, and eGFR. In each case, the model is not adjusted for the stratification variable.

### 4.8 Results of TyG-WWI change groups

The participants were divided into two classes based on changes in the TyG-WWI from 2011 to 2015 via K-means cluster analysis (Supplementary Figure S1). Class 1 was defined by stable, relatively low TyG-WWI values, whereas Class 2 was characterized by relatively high TyG-WWI values (Supplementary Table S4). Multivariate logistic regression analysis revealed a significant association between the TyG-WWI change group and stroke risk. Compared with Class 1 participants, Class 2 participants had a greater risk of stroke across all the models (Model I: OR = 1.70, 95% CI 1.43–2.02, *p* < 0.0001; Model II: OR = 1.71, 95% CI 1.42–2.05, *p* < 0.0001; Model III: OR = 1.30, 95% CI 1.06–1.61, *p* = 0.0124).

## 5 Discussion

### 5.1 Summary of the main findings

In this study on the link between TyG-WWI and stroke risk, we found that stroke risk rose significantly with higher TyG-WWI quartiles. In Model III, compared to Q1, Q2, Q3, and Q4 showed a progressive increase in HR, which were 1.35 (95% CI 1.08–1.69, *p* = 0.0094), 1.38 (95% CI 1.09–1.73, *p* = 0.0069), and 1.42 (95% CI 1.09–1.85, *p* = 0.0086) respectively, and the P for trend was statistically significant at 0.0158. Two-piecewise linear regression model analysis showed an inflection point of TyG-WWI at 43.32. Left of it, TyG-WWI wasn’t significantly associated with stroke risk (HR = 0.99, 95% CI 0.97–1.01, *p* = 0.2153); right of it, the association became significant (HR = 1.03, 95% CI 1.01–1.05, *p* = 0.0008). Stratified analysis (Table 5) indicated a significant interaction for hypertension status (P for interaction = 0.0493), with the strongest association in non-hypertensive individuals (HR = 1.94, 95% CI: 1.29–2.91, *p* = 0.0014 in Q4). Other variables did not show significant interactions. In conclusion, TyG-WWI is significantly and dose—dependently associated with stroke risk, particularly in non-hypertensive individuals.

### 5.2 Comparison with previous studies

Prior studies have demonstrated the associations of the triglyceride-glucose (TyG) index and its combinations with obesity-related metrics (e.g., TyG-WWI, TyG-BMI, TyG-WC) with cardiovascular disease (CVD) risk and mortality. For instance, TyG has been shown to correlate with increased CVD risk (50) and has potential value in predicting CVD and its mortality (51, 52). Similarly, the weight-adjusted waist index (WWI) has been linked to stroke risk in a linear manner, with higher WWI associated with increased stroke risk in Chinese adults aged ≥45 years (53), and it also exhibits potential in assessing all-cause and cardiovascular mortality (34).

Our study extends these findings by combining TyG and WWI to predict future stroke risk. A prior study using the CHARLS database reported no significant association between baseline TyG-WWI and CVD risk (30), whereas our results indicate a significant association between baseline TyG-WWI and incident stroke. The inconsistency may stem from differences in inclusion criteria or endpoint definitions.

Investigating the non-linear relationship between TyG-WWI and stroke is important because metabolic risk factors often exhibit threshold effects rather than simple linear patterns. TyG-WWI reflects both insulin resistance and abnormal fat distribution, and their combined impact on vascular health may accelerate once a critical level is reached. Linear models may overlook such inflection points and underestimate risk in high-exposure subgroups. By modeling non-linearity, we can better identify high-risk individuals, reveal potential saturation or threshold effects, and improve the precision of risk stratification in clinical settings. Our findings support this, showing a clear non-linear pattern with an inflection point at 43.32, beyond which stroke risk increases more steeply. This contrasts with previous work on TyG-BMI, highlighting how different anthropometric indices may capture metabolic risk differently across populations (32). Both studies identified non-linear relationships, but at different inflection points (43.32 for TyG-WWI vs. 174.63 for TyG-BMI). In our study, TyG-WWI showed a significant interaction with hypertension status on stroke risk, with a stronger link in non-hypertensives. This may be as metabolic abnormalities reflected by TyG-WWI more directly drive stroke risk in non-hypertensives, while hypertension—related vascular damage and treatments might mask this effect in hypertensives.

In addition, cumulative TyG-related indices have been shown to correlate with CVD risk. A study found that cumulative TyG-WWI was significantly associated with CVD risk (30). Another study reported that a 1 SD increase in cumulative mean TyG-BMI was associated with a 1.168-fold increase in CVD risk (35). Furthermore, research focusing on the cumulative impact of TyG-related index changes from 2012 to 2015 revealed that changes in TyG-WC were significantly associated with CVD risk, with the strongest associations observed in higher change categories and cumulative indices (54). These findings are consistent with our results, which indicate that higher changes in TyG-related indices are associated with increased incident stroke risk.

### 5.3 Potential mechanisms

Although the exact mechanisms are unclear, TyG-WWI may be associated with insulin resistance (22, 55, 56). Insulin resistance impairs glucose and lipid metabolism, leading to compensatory hyperinsulinemia and increased free fatty acid levels (57). Chronic low-grade inflammation, characterized by elevated proinflammatory cytokines from adipose tissue, exacerbates insulin resistance and promotes oxidative stress, which further damages cells and reduces nitric oxide bioavailability (58, 59). Adipose tissue dysfunction, particularly in visceral fat, results in the secretion of adipokines such as leptin and adiponectin, which contribute to metabolic disturbances (60, 61). Dyslipidemia, which is characterized by elevated triglycerides and low HDL, results from increased hepatic triglyceride synthesis and reduced HDL production (62). Hypertension arises from insulin resistance-induced sodium retention and increased vascular stiffness. Endothelial dysfunction, caused by impaired nitric oxide signaling, leads to vasoconstriction and vascular inflammation (63, 64). These mechanisms collectively create a cycle that worsens metabolic abnormalities and increases cardiovascular risk.

In our study, the significant association between the TyG-WWI and stroke risk, particularly above the identified inflection point, likely reflects the combined impact of these metabolic derangements. The nonlinear relationship observed, with a greater risk increment at higher TyG-WWI levels, underscores the concept that beyond a certain threshold, the metabolic burden poses a more substantial threat to cerebrovascular health. This threshold effect may be attributed to the progressive dysfunction of metabolic regulatory mechanisms and the overwhelming compensatory pathways that maintain vascular homeostasis.

### 5.4 Limitations and future directions

Our study has several limitations. Firstly, the inherent limitations of an observational study design preclude the exclusion of unmeasured confounding. Even after accounting for known potential confounders, residual confounding due to unmeasured or uncontrolled factors may remain. Secondly, self-reported physician diagnosis is used to measure stroke incidence, which may be subject to recall bias or misclassification. However, prior validation studies suggest that such self-reports can be sufficiently accurate in large-scale cohorts. For example, the China Kadoorie Biobank reported an overall accuracy of 90% for self-reported strokes (65). Similarly, the Health and Retirement Study in the U. S. found that self-reported strokes provided incidence rates and risk factor associations comparable to those from clinically verified data (66). Thirdly, measurement errors in assessing TyG-WWI components could affect the results. Furthermore, the applicability of the findings to younger cohorts and diverse ethnic groups may be limited, given the study’s focus on middle-aged and elderly individuals from China. Finally, the observational nature of this study limits its ability to establish causal relationships between TyG-WWI and stroke risk, as it can only identify an association rather than causation. Future research should extend follow-up periods to confirm long-term impacts and explore diverse populations for generalizability. Intervention studies are needed to determine whether reducing the TyG-WWI lowers stroke risk. Further investigations into the biological mechanisms involved and their combined effects on other risk factors are also warranted. Finally, cost-effectiveness analyses using the TyG-WWI for stroke risk assessment in various healthcare settings would be valuable.

## 6 Conclusion

This national prospective cohort study demonstrates that baseline TyG-WWI independently predicts incident stroke in middle-aged and elderly Chinese adults. Notably, we observed a nonlinear association with a threshold effect at TyG-WWI = 43.32. Below this inflection point, TyG-WWI showed no significant association with stroke risk, whereas beyond it, risk increased progressively with higher TyG-WWI values. The impact of TyG-WWI on stroke risk appears to be more pronounced in non-hypertensive individuals.

## Data Availability

The datasets presented in this study can be found in online repositories. The names of the repository/repositories and accession number(s) can be found at: https://charls.pku.edu.cn/.

## Footnotes

^1^ https://charls.pku.edu.cn

## Glossary

TyG-WWI: Triglyceride glucose-waist adjusted waist index
CHARLS: China Health and Retirement Longitudinal Study
CLD: Chronic lung disease
CKD: Chronic kidney disease
HDL-c: Serum high-density lipoprotein cholesterol
LDL-c: Low-density lipoprotein cholesterol
TC: Total cholesterol
HGB: Hemoglobin concentration
CRP: C-reactive protein
WBC: White blood cell count
PLT: Platelet
HBA1c: Hemoglobin A1c
CYSC: Cystatin C
UA: Uric acid
BUN: Blood urea nitrogen
CREA: Creatinine
eGFR: Estimated glomerular filtration rate
FBG: Fasting blood glucose
TG: Triglycerides
BMI: Body mass index
SBP: Systolic blood pressure
DBP: Diastolic blood pressure
