# Supplementary material for: Association between triglyceride glucose-waist-adjusted waist index and incident stroke in Chinese adults: a prospective cohort study
Source: Front Nutr. 2025 Jul 1;12:1612864. doi: 10.3389/fnut.2025.1612864 (PMC12259417; doi:10.3389/fnut.2025.1612864)
Supplement: Supplementary file 1 [file Table_1.docx]

**Table S1 Collinearity screening:**

|  | Step 1 |
| --- | --- |
| Age | 1.6 |
| Gender | 2.5 |
| Drinking status | 1.5 |
| Smoking status | 1.7 |
| Cancer | 1 |
| CLD | 1 |
| CKD | 1 |
| Hypertension | 1.1 |
| Diabetes | 1.4 |
| HDL-c | 1.3 |
| LDL-c | 3.2 |
| TC | 3.6 |
| HGB | 1.2 |
| CRP | 1.1 |
| WBC | 1.2 |
| PLT | 1.1 |
| HBA1C | 1.3 |
| CYSC | 1.6 |
| UA | 1.6 |
| eGFR | 2.1 |
| TyG-WWI | 1.5 |

HDL-c high-density lipoprotein cholesterol, LDL-c low-density lipoproteins cholesterol, TC total cholesterol, HGB hemoglobin concentration, CRP C-Reactive Protein, WBC White Blood Cell in Thousands, PLT platelets, HBA1c hemoglobin A1c, CYSC Cystatin C, UA Uric Acid, BUN Blood Urea Nitrogen, CREA Creatinine, eGFR Estimated glomerular filtration rate, FBG Fasting Blood Glucose, TG Triglycerides, BMI Body Mass Index, CLD Chronic Lung Diseases, CKD Chronic kidney diseases, , TyG-WWI triglyceride glucose-weight-adjusted waist index

**Table S2. Factors influencing the risk of stroke were analyzed by univariate Cox proportional hazards regression.**

|  | Subject characteristics | (HR.,95%CI) | *P* |
| --- | --- | --- | --- |
| Age | 59.32 ± 9.22 | 1.03 (1.03, 1.04) | <0.0001 |
| Sex |  |  |  |
| Female | 4816 (53.72%) | Ref |  |
| Male | 4149 (46.28%) | 1.11 (0.97, 1.27) | 0.1249 |
| Drinking status |  |  |  |
| Never drinker | 5484 (61.17%) | Ref |  |
| Ever drinker | 727 (8.11%) | 1.49 (1.19, 1.86) | 0.0006 |
| Current drinker | 2754 (30.72%) | 1.07 (0.92, 1.25) | 0.3683 |
| Smoking status |  |  |  |
| Never smoker | 5484 (61.17%) | Ref |  |
| Ever smoker | 2712 (30.25%) | 1.12 (0.96, 1.31) | 0.1349 |
| Current smoker | 769 (8.58%) | 1.63 (1.31, 2.02) | <0.0001 |
| Cancer |  |  |  |
| No | 8889 (99.15%) | Ref |  |
| Yes | 76 (0.85%) | 0.76 (0.31, 1.82) | 0.5357 |
| CLD |  |  |  |
| No | 8101 (90.36%) | Ref |  |
| Yes | 864 (9.64%) | 1.14 (0.91, 1.43) | 0.2535 |
| CKD |  |  |  |
| No | 8454 (94.30%) | Ref |  |
| Yes | 511 (5.70%) | 1.38 (1.06, 1.79) | 0.0151 |
| Hypertension |  |  |  |
| No | 5377 (59.98%) | Ref |  |
| Yes | 3588 (40.02%) | 2.48 (2.16, 2.84) | <0.0001 |
| Diabetes |  |  |  |
| No | 7463 (83.25%) | Ref |  |
| Yes | 1502 (16.75%) | 1.41 (1.20, 1.67) | <0.0001 |
| HDL-c | 51.27 ± 15.33 | 0.99 (0.98, 0.99) | <0.0001 |
| LDL-c | 116.50 ± 35.20 | 1.00 (1.00, 1.00) | 0.0178 |
| TC | 193.94 ± 38.77 | 1.00 (1.00, 1.00) | 0.0096 |
| HGB | 14.38 ± 2.20 | 1.05 (1.02, 1.08) | 0.0012 |
| CRP | 2.62 ± 7.09 | 1.01 (1.01, 1.02) | 0.0001 |
| WBC | 6.24 ± 1.88 | 1.05 (1.02, 1.09) | 0.0025 |
| PLT | 211.87 ± 72.98 | 1.00 (1.00, 1.00) | 0.0090 |
| HBA1c | 5.27 ± 0.80 | 1.22 (1.14, 1.30) | <0.0001 |
| CYSC | 1.00 ± 0.27 | 1.69 (1.41, 2.02) | <0.0001 |
| UA | 4.45 ± 1.25 | 1.10 (1.04, 1.16) | 0.0006 |
| eGFR | 96.89 ± 15.52 | 0.98 (0.98, 0.99) | <0.0001 |
| TyG-WWI | 51.91 ± 7.72 | 1.04 (1.03, 1.05) | <0.0001 |

Abbreviations: SD, standard deviation; N, number; LDL-c, low-density lipoproteins cholesterol; BUN, blood urea nitrogen; TyG-BMI, Triglyceride glucose-body mass index; WBC, white blood cell count ; PLT, platelet; BMI, body mass index; HGB, hemoglobin concentration; UA, Uric acid; TC, total cholesterol; HBA1c, hemoglobin A1c; DBP, diastolic blood pressure; TG, triglyceride; eGFR, Estimated glomerular filtration rate; CLD, Chronic Lung Diseases; DM, diabetes mellitus; Scr, serum creatinine; HDL-c, high-density lipoprotein cholesterol; CKD, Chronic kidney diseases; SBP, systolic blood pressure, TyG-WWI triglyceride glucose-weight-adjusted waist index

HR, hazard ratio; Ref: reference; CI: confidence.

**Table S3 Association of TG, FPG, TyG and WWI with stroke risk in different models.**

|  | Model | (HR.,95%CI) | *P* |
| --- | --- | --- | --- |
| TG | a | 1.0 (1.0, 1.0) | 0.622 |
| TyG | b | 2.04 (1.37, 3.04) | 0.0005 |
| FBG | c | 1.00 (1.00, 1.00) | 0.3340 |
| WWI (kg/m2) | d | 1.03 (0.97, 1.10) | 0.2723 |

Model a: we adjusted age, gender, drinking status, smoking status, cancer, CLD,CKD, hypertension， diabetes， HDL-c, LDL-c, TC, HGB, CRP, WBC, PLT, HBA1c, CYSC, UA, eGFR, FBG, WWI.

Model b: we adjusted age, gender, drinking status, smoking status, cancer, CLD,CKD, hypertension， diabetes， HDL-c, LDL-c, TC, HGB, CRP, WBC, PLT, HBA1c, CYSC, UA, eGFR, WWI.

Model c: we adjusted age, gender, drinking status, smoking status, cancer, CLD,CKD, hypertension， diabetes， HDL-c, LDL-c, TC, HGB, CRP, WBC, PLT, HBA1c, CYSC, UA, eGFR, WWI, TG.

Model d: we adjusted age, gender, drinking status, smoking status, cancer, CLD,CKD, hypertension， diabetes， HDL-c, LDL-c, TC, HGB, CRP, WBC, PLT, HBA1c, CYSC, UA, eGFR, TYG.

HR, hazard ratio; CI: confidence

**Table S4 Multivariate logistic regression analysis of the association between different TyG-BMI change groups (change from 2011 to 2015) and stroke risk**

|  | Model I  (OR., 95%CI) *p* | Model II  (OR., 95%CI) *p* | Model III  (OR., 95%CI) *p* |
| --- | --- | --- | --- |
| Class 1 | Ref | Ref | Ref |
| Class 2 | 1.70 (1.43, 2.02) <0.0001 | 1.71 (1.42, 2.05) <0.0001 | 1.30 (1.06, 1.61) 0.0124 |

Model I: we did not adjust other covariates.

Model II: we adjust age and gender.

Model III: we adjust age, gender, drinking status, smoking status, cancer, CLD, CKD, hypertension, diabetes, HDL, LDL, TC, HGB, CRP, WBC, PLT, HBA1c, CYSC, UA, eGFR.

**Fig S1 The K-means cluster analysis divided participants into two distinct groups based on changes in TyG-WWI from 2011 to 2015.**


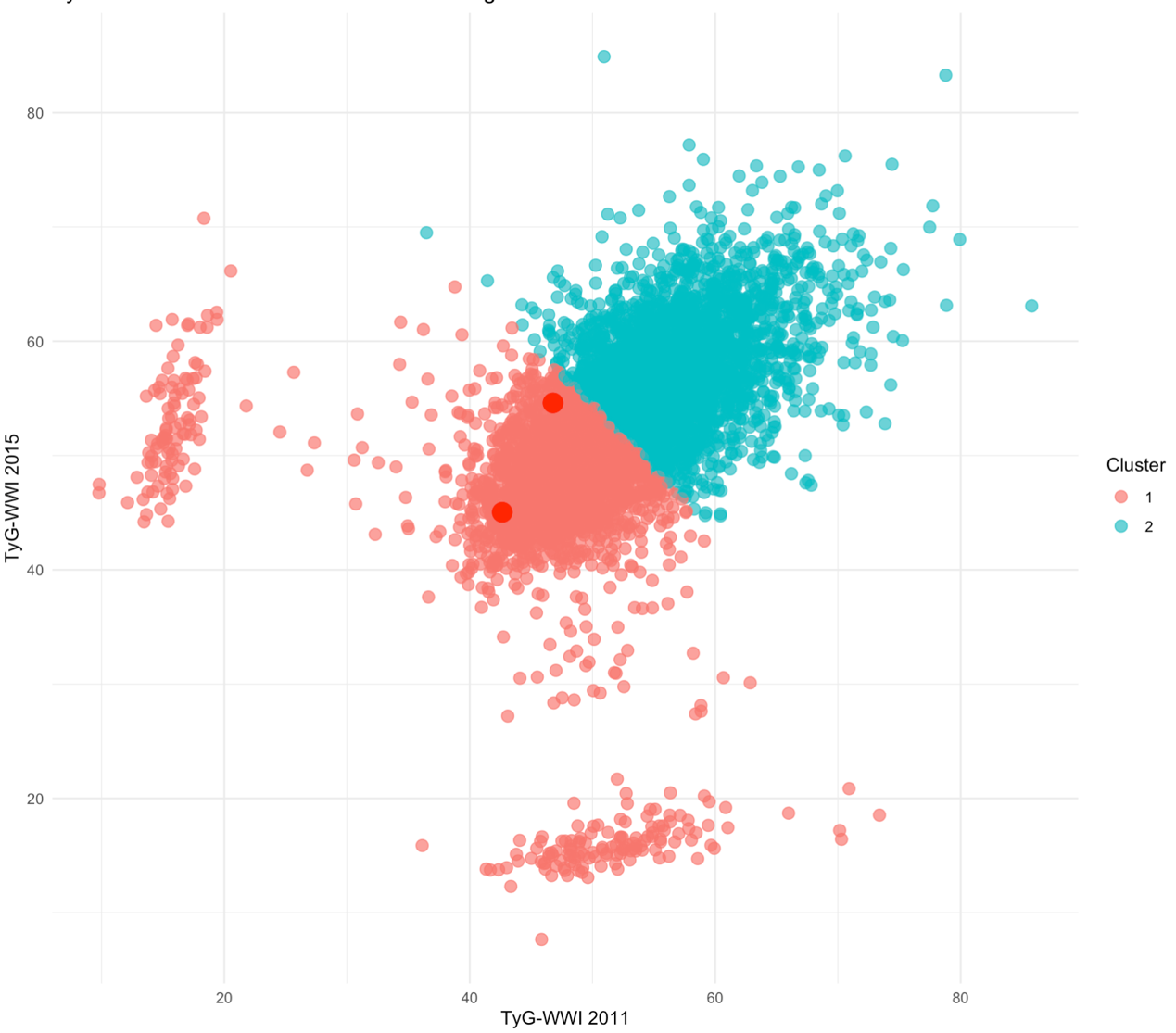


The x-axis shows the TyG-WWI values for 2011, while the y-axis shows the values for 2015. Each point represents a participant, with colors indicating two clusters: red (Cluster 1) for the low TyG-WWI group and blue (Cluster 2) for the high TyG-WWI group. Solid dots mark the cluster centers, reflecting the average TyG-WWI levels of each group. Most participants’ TyG-WWI values remained relatively stable over these four years, with a clear distinction between the two groups. Cluster 1 is mainly in the lower-left area (low values), and Cluster 2 is in the upper-right area (high values).
